# Letter from Dr. Herrick

**Published:** 1847-02

**Authors:** William B. Herrick

**Affiliations:** In Camp, near Mon Clova, Mexico


### ARTICLE VII.

In Camp, near Mon Clova, Mexico,
November 5, 1846.

I am now writing in the vicinity of a Mexican town, containing about 5000 inhabitants, situated between, and nearly surrounded by, mountains.

The distance from San Antonio, Texas (the point where the column under General Wool was organized), is about 400 miles. We started from San Antonio the second day of October, and arrived here the 2d of November, thus making a march of thirty days without stopping.

This marching, day after day, so far into the interior of Mexico without meeting with any opposition, and with no prospect of a fight, causes a great deal of dissatisfaction, and is the subject of constant complaint with our Illinois Volunteers.

When on the march, we start in the morning by sunrise, go from 12 to 15 miles without halting, excepting for a short time now and then, and encamp for the rest of the day and following night, generally about 11 o'clock, A. M. As I remarked in a former letter, we pass through all the towns in our way with drums beating and colors flying. In the towns of most importance we take formal possession, and plant the American Flag in the most public and conspicuous position.

A few miles from each town we have, in every instance so far, been met by the Alcalde, or ruler, with the principal men of the place, as a deputation from the citizens; and it is by means of such deputations that the people throughout this section of country, have unanimously, as I believe, expressed their determination to offer no opposition to our progress. In truth, it seems to be their wish, not only to remain neutral in the contest between the United States and their central government, but many of them openly express a desire to become separated from it, and a wish to form a republic of their own, or to come under the protection of that of the United States.

As an evidence of their oppressed condition, and to show from whence this feeling originates, it may be stated that, according to their representation, the Mexican army, in marching through their country, would live by robbing and plundering the inhabitants. The officers of ours, on the other hand, treat them as friends when they are friendly, and pay them fair prices for the means of subsistence.

With regard to the question, will it be good or bad policy to admit this portion of Mexico into the Union? intelligent men will of course differ in opinion. For my own part, I do not believe the Mexicans, as a mass, are, as yet, sufficiently far advanced in civilization and intelligence to admit of their establishing and sustaining a truly republican form of government; and therefore it would be not only bad policy, but dangerous, to endow them, at once, with the rights and privileges of citizens of the United States.

As examples of their barbarous customs, and tyrannical laws, the following may serve as specimens.

In many parts of the country matrimonial engagements are temporary merely: it being a common custom, as I am informed, for parties to agree to live together as man and wife for a few months only, at the end of which time either is at liberty to dissolve the compact. As an excuse for this demoralizing custom, they say that the Indians have, in many parts of the country, destroyed so many of the men, that there are to every male inhabitant five or six females, and that unless such an indulgence be permitted, the population of their towns will rapidly diminish, and eventually become entirely extinct. Whether this be a sufficient reason or not for such an absurd and ridiculous custom, our readers can, for themselves, determine.

One example of their oppressive laws, and we are done for this time with Mexican institutions, and the character of the people.

In marching through this part of Mexico, we find the inhabitants, not scattered over the country as in the United States, but collected together, in towns from 20 to 50 miles apart, in the rich valleys between the mountains. We frequently meet with a collection of rude buildings surrounded by a wall, and inhabited by numerous slaves who, like the herds of cattle, and many square leagues of land in the vicinity, are owned by a single tyrant. An establishment of this kind is called by the Mexicans, a hacienda.

The slaves thus condemned to servitude and a life of bondage are not, like ours of the United States, marked by nature as a different race of beings from their masters, but in many cases, the Mexican slave is as well formed, physically, and is as intelligent as the tyrant who owns and governs him.

This being the case, the question naturally arises, why is it that people of the same race, not differing materially in natural endowments, are a few of them masters and the rest slaves?

This state of society results from a most tyrannical law, which provides that whenever one person becomes indebted to another, the debtor, unless he is able to make immediate payment, becomes at once the slave of the creditor, and is obliged to labor for him at a rate not exceeding 3 or 4 dollars per month, till the demand is cancelled. The amount earned, in this way, by the debtor, is often less than the sum required for his subsistence, consequently he is obliged to purchase on credit still more of his master, and thus to perpetuate a life of bondage.

The worst feature in this system of slavery for debts is, doubtless, that which provides for the perpetuation of this life of bondage; for it is not the debtor alone that is bound thus to give up his liberty, but his children inherit his debts, and with them loose the rights of free men.

Having thus given a few hints concerning the customs and laws of the Mexicans, we will now proceed to the consideration of a subject which more immediately interests the people of Illinois, especially the medical men of our State.

Since joining the army, I have, for the most part of the time, been the only medical officer attached to the 1st regiment of Illinois Volunteers, and can of course speak with entire confidence, with regard to the diseases which have prevailed among the volunteers of this regiment.

On entering upon the duties of my office at Camp Crocket, near San Antonio, Texas, I found from 60 to 70 on the sick report. Of the cases thus reported, a majority were miasmatic fevers of a mild grade, which yielded readily to gentle laxatives and quinine, in doses of from 10 to 15 grains in the 24 hours.

This kind of treatment soon reduced the number of sick from 75 to 40, in a command of about 800 men. My experience, so far, in the army, with this class of diseases, has fully confirmed my belief in the utility of administering large doses of this most efficient remedy in miasmatic diseases. I have given it in all stages of these fevers, with uniform success, and without, at any time, producing unfavorable results. Diarrhœa is another disease very prevalent in the army; but, in most cases, it is of a mild form and yields readily to some mild mercurial, such as blue pill followed by, or combined with, opium and camphor. There are a few cases of a chronic form however, that do not yield so readily, but continue obstinately to debilitate the patients and produce emaciation. Many of these, I have no doubt, are dependent upon an ulcerated condition of the mucous membrane, whilst others perhaps result from torpid or diseased livers.

But of all the diseases to which the volunteers have been subject, those of the lungs consequent upon measles, have been the most destructive to life. From what I could learn of the diseases, previous to my joining my regiment, nine-tenths of those which proved fatal were of the lungs, and in all the cases, the patients had had measles whilst on the march, or in camp, in cold tents and sleeping upon the ground. To those acquainted with the progress of this cutaneous disease, and its tendency to produce lung affections under any circumstances, it will not appear strange that it was the cause of fatal results in so many cases, nor will they be surprised to learn, that all kinds of treatment under the circumstances proved of little or no avail.

We are now on our march through the high lands of Mexico, where there are, probably, as few causes of disease, as in any section of the world. It is to be expected, therefore, that unless we get into a brush with the Mexicans, and as a consequence, have a few surgical cases, we shall have but little of professional interest to communicate. Still, we shall not let any opportunity pass, of giving to our readers interesting intelligence, whether it be of a professional or general nature.

WILLIAM B. HERRICK.

